# An equity indicator for assessing mental healthcare access: a national population case study

**DOI:** 10.1017/S2045796024000738

**Published:** 2024-11-29

**Authors:** S. Dawadi, F. Shawyer, E. Callander, S. Patten, B. Johnson, S. Rosenberg, V. Lakra, E. Lin, H. Teede, G. Meadows, J. Enticott

**Affiliations:** 1 Monash Centre for Health Research and Implementation, Monash University, Clayton, VIC, Australia; 2 Southern Synergy Department of Psychiatry, Monash University, Melbourne, VIC, Australia; 3 School of Public Health, University of Technology Sydney, Sydney, NSW, Australia; 4 Cumming School of Medicine, Department of Health Sciences, University of Calgary, Calgary, AB, Canada; 5 Brain and Mind Centre, Sydney Medical School, University of Sydney, Sydney, NSW, Australia; 6 Health Research Institute, University of Canberra, Canberra, ACT, Australia; 7 Mental Health Services, Northern Health, Melbourne, VIC, Australia; 8 Department of Psychiatry, The University of Melbourne, Melbourne, VIC, Australia; 9 Department of Psychiatry, University of Toronto, Toronto, ON, Canada; 10 ICES (Formerly the Institute for Clinical Evaluative Sciences), Toronto, ON, Canada; 11 Centre for Mental Health and Community Wellbeing, School of Population and Global Health, University of Melbourne, Melbourne, VIC, Australia; 12 School of Primary and Allied Healthcare, Monash University, VIC, Australia; 13 Monash Health, Dandenong, VIC, Australia

**Keywords:** equity, health system indicators, mental health services, needs based

## Abstract

**Aims:**

Achieving equitable healthcare access is a global challenge. Improving whole-population mental health and reducing the global burden of mental disorders is a key recommendation of the 2018 Lancet Global Mental Health Commission, which proposed monitoring national indicators, including the proportion of people with severe mental disorders who are service-users. This study aims to derive an equity indicator from national datasets integrating need, service utilisation and socioeconomic status, and demonstrate its utility in identifying gaps in mental health service use amongst those with the greatest need, thereby guiding equitable healthcare delivery.

**Methods:**

We present a case study of a universal health insurance scheme (Medicare) in Australia. We developed the equity indicator using three national datasets. Geographic areas were linked to an area-based socioeconomic deprivation quintile (Census 2016). Per geographic area, we estimated the number with a mental healthcare need using scores ≥30 on the Kessler-10 (Australian National Health Surveys 2015 and 2018), and obtained the number of services used, defined as mental health-related contacts with general practitioners and mental health professionals (Medicare administrative data 2015–2019). We divided the number of services by the population with an estimated mental healthcare need and averaged these use-rates across each socioeconomic deprivation quintile. The equity indicator is the ratio of the use-rates in the least versus most deprived quintiles.

**Results:**

Those estimated to have the greatest need for mental healthcare in 2019 ranged between 8.2% in the most disadvantaged area quintile (Q1) and 2.4% in the least (Q5), corresponding to a proportional increase of 27.7% in Q1 and 19.5% in Q5 since 2015. Equity-indicator-adjusted service rates of 4.2 (3.8–4.6) and 23.9 (22.4–25.4) showed that individuals with the highest need for care residing in Q1 areas received a stark 6 times fewer services compared to their Q5 counterparts, producing an equity indicator of 6.

**Conclusions:**

As the global prevalence of common mental disorders may be increasing, it is crucial to calculate robust indicators evaluating the equity of mental health service use. In this Australian case study, we developed an equity indicator enabling the direct comparison of geographic areas with different need profiles. The results revealed striking inequities that persisted despite publicly-funded universal healthcare, recent service reforms and being a high-income country. This study demonstrates the importance and feasibility of generating such an indicator to inform and empower communities, healthcare providers and policymakers to pursue equitable service provision.

## Introduction

Common mental disorders, including depressive and anxiety disorders, are leading contributors to global disability, impacting over one billion people in 2018 (GBD 2016 Disease and Injury Incidence and Prevalence Collaborators, 2017). Studies typically, though not universally, have found increasing prevalence in the past two decades (Beller *et al.*, 2021; Moreno-Agostino *et al.*, 2021). Since then, estimates suggest further growth, compounded by events associated with the COVID-19 pandemic (Enticott *et al.*, 2022; World Health Organization, 2022).

Efforts at mental healthcare reform have occurred globally for many decades. While the scale and nature of these reforms varies greatly between – and sometimes within – countries, general trends include de-institutionalisation, increased community-based care, stigma reduction, increased policy co-design with service users, development of lived experience workforce and calls for adequate funding and workforce capacity (Rosen *et al.*, 2022). However, these efforts have had limited effects on the population level burden of mental disorders, even within high-income countries (Chandra and Chand, 2018). Key reasons given for this are treatment and quality of care gaps, with quality treatment not accessed by all who need it, especially people within lower socioeconomic groups or residing in deprived areas (Chandra and Chand, 2018; World Health Organization, 2014). This socioeconomic disparity is reported even in countries and regions with ‘universal’ healthcare, including Canada (Bartram and Stewart, 2019), Europe (Pinto-Meza *et al.*, 2013) and Australia (Meadows *et al.*, 2015; Pirkis *et al.*, 2022). However, in one UK study, between 2005 and 2009, services were increasingly concentrated in lower incomes for public, but not private psychologists (Jokela *et al.*, 2013), suggesting that such disparities are not inevitable.

Improving the mental health of whole populations and reducing the global burden of mental disorders is a key recommendation made by the 2018 Lancet Commission on global mental health (Patel *et al.*, 2018). To help close the treatment gap, the Commission proposed the reporting and monitoring of indicators such as proportions of people with severe mental disorders who are using services (Chandra and Chand, 2018). However, identifying people in the community with mental illness who don’t use services is challenging, therefore making such indicators difficult to operationalise. Instead, service usage rates are commonly reported, but these rates alone fail to reveal if the services are being accessed by those in need.

Indicators to assess mental healthcare equity, access and benefit for target populations, are therefore vital to health service monitoring. Indicators should be devised using a good representative sample of the health needs of people in different geographical areas, such as national health surveys (Hashmi *et al.*, 2023; Radinmanesh *et al.*, 2021). Then, adjusting service-use rates for socioeconomic area-based need allows for more direct comparisons across different areas (Bartram and Stewart, 2019). While there are regional service planning examples (Barr *et al.*, 2014; Kirigia, 2009; Meadows and Singh, 2003; Rush *et al.*, 2019), no studies have integrated national data on needs, service use and socioeconomic area status in Australia or to our knowledge globally. In evaluations of mental health service use over geographic areas, when included, socioeconomic status or proxy measures were most often used as a variable to stratify findings by (Blais *et al.*, 2003; Levinson *et al.*, 2006; Muhajarine *et al.*, 2012), or as a covariate in regression models (Bayoumi *et al.*, 2020; McBain *et al.*, 2022; Tibaldi *et al.*, 2005), or in concentration indexes and horizontal equity indexes (Fan *et al.*, 2022). None presented real-world service-use rates adjusted for the socioeconomic variation in health service need, and allowing for comparisons between areas. Several studies described existing or potential resource distribution formulae (Johnston *et al.*, 2021; Radinmanesh *et al.*, 2021); some of which incorporated socioeconomic status or a proxy measure. Several studies used data or examples from low and middle income countries (Love-Koh *et al.*, 2020; World Health Organization, 2008).

Our aim was to create a national mental healthcare equity indicator that integrates need, service utilization and socioeconomic status to guide equitable mental healthcare delivery. This research addresses vital gaps, bringing the potential to provide global policymakers with an indicator to assess the impact of mental health reforms on their target populations. Here we present a case study from Australia, where federally-funded mental health services (Box 1) are universally subsidized through Medicare, aiming to provide accessible care to those in need. Using the equity indicator, we aim to assess whether service use-rates vary amongst individuals with the highest level of estimated need across the different areas of Australia.

Box 1. A National Case Study: Australia

**Australian mental disorder prevalence**: Similar to other high-income nations (Pinto-Meza *et al.*, 2013), socioeconomic inequality exists in Australia, with higher mental health condition rates in deprived areas compared to affluent ones. In 2016, diagnosed anxiety, affective and substance use disorders had a 24% prevalence in the most disadvantaged area quintile and 17% in the least disadvantaged quintile. Highly elevated psychological distress, indicative of severe mental illness, was 4.1% in the most disadvantaged area quintile and 1.0% in the least disadvantaged quintile (Enticott *et al.*, 2016). Recent Australian National Health Survey data suggests this disparity persists and may be growing (Enticott *et al.*, 2022).

**Mental health expenditure**: Mental health reforms have taken place in Australia over the past three decades, with mental health spending exceeding AUD$11.6 billion. However, the proportion of mental health costs in the overall health budget has remained unchanged since 1992 (Australian Institute of Health and Welfare, 2023b).

**Australian healthcare**: Federally funded mental health services are universally subsidized through the Medicare-subsidised scheme. Annual Medicare service usage data shows an increase, from 6% of Australians receiving at least one Medicare-subsidised mental health service in 2009/10 to 10% in 2019/20 (Australian Institute of Health and Welfare, 2023b). Medicare provides mental health services through general practitioners (GPs) and other private providers, including, psychologists, allied-health professionals and psychiatry services. While some costs are subsidized, out-of-pocket expenses have been rising (Rosenberg *et al.*, 2022).

Medicare mental health services are distinct from community mental health services, which are state-funded and consist of treatment provided in the community and hospital-based outpatient care. Community mental health services have a high entry-threshold, typically treating a client-base with very serious mental illness at the times when illness suddenly worsens, and complex clients (Cook, 2019). With often high caseloads, support to this group may be limited by time with limited workforce and resources. Community mental health services are therefore not a ‘replacement’ for Medicare-subsidised mental health, as this creates a two-tiered healthcare system contrary to the principle of universality, especially considering that the two service types are substantially different (see Supplementary file 1).

**Evaluation**: Evaluations of Medicare-subsidised mental health services in Australia have explored whether those in need access them. The first nationwide Medicare data evaluation from 2007/08 to 2009/10 reported minimal socioeconomic differences in service utilization (Pirkis *et al.*, 2022). However, later analyses for the period of 2007/08 to 2010/11 found greater socioeconomic inequity in access to psychiatrist-provided services, with 45/1000 people in the most disadvantaged area quintile and 117/1000 people in the least disadvantaged quintile (Meadows *et al.*, 2015). Another evaluation from 2018 to 2021 confirmed these inequities in services, with individuals with lower incomes missing out despite higher levels of need (Pirkis *et al.*, 2022).

**Utility of a needs-weighted area-based indicator**: Previous national-level evaluations reported service use-rates by area, ignoring specific mental health needs in various socioeconomic areas. Service use-rates alone may underestimate inequities. A recent analysis adjusted for area-based needs, indicating a significant increase in inequitable and unmet needs in Australian psychiatric care utilization between 2009 and 2017; however this study used a sample (i.e. not a data set of national service data) (Hashmi *et al.*, 2023).

## Methods

As an exemplar case study, we developed the mental health equity indicator within the Australian health system. Box 1 shows contextual information about Australian mental health services and policy. The equity indicator was generated from three high-quality, publicly-available national datasets with information on healthcare need, service use and area-based socioeconomic status (Table 1). The equity indicator was designed to enable direct comparison between areas with different need profiles; Table 2 and the detailed supplementary file describe indicator calculation.

**Table 1.** Indicator data sources applied to each year of service data. Also shown are the percentages estimated to have the greatest need for mental healthcare within each Index of Relative Socio-economic Disadvantage (IRSD) area quintile

National Health Survey^a^		Census 2016 and forward estimates^b^		Mental health (Medicare) service data^c^
Percentages estimated to have greatest need for mental healthcare (very-high level Kessler − 10 psychological distress; in 18–64 yo)		IRSD quintile (1 most disadvantaged area; 5 least disadvantaged area)	# Residing in each IRSD quintile in each year	Service used in each year by provider type
2014/15	6.28	IRSD1	2015 & 2016	2015 & 2016
	4.05	IRSD2		
	3.73	IRSD3		
	2.89	IRSD4		
	2.00	IRSD5		
2017/18	8.02	IRSD1	2017, 2018 & 2019	2017, 2018 & 2019
	5.55	IRSD2		
	3.03	IRSD3		
	3.19	IRSD4		
	2.39	IRSD5		

Equity indicator adjusted service rates are calculated by: number of services utilized divided by the sub-population with need for mental healthcare. The equity indicator was developed through linkage of three national, publicly available data sources:

a National Health Surveys.

b Census 2016 derived IRSD area quintiles and population estimates by areas calculated by the Australian Bureau of Statistics.

c Mental health service data.

**Table 2.** Equity indicator. Estimated population and prevalence of those with greatest need for mental healthcare in Index of Relative Socio-economic Disadvantage (IRSD) area quintile 1 (most disadvanted) and quintile 5 (least disadvanted). See supplementaly files for quintiles 2–4

Year	IRSD quintile areas	C. Population	D. Percent with very-high psychological distress	E. Estimated number with greatest need for mental healthcare	F. Proportional estimated increase (percent) in those with greatest need for mental healthcare	G. All service use rate (per capita)	H. Equity-indicator-adjusted service rate	I. Equity indicator (Ratio IRSD 5:1)*
2015	Q1	3,904,729	6.28%	245,217	82,133 (27.71%)	0.32	5.09	–
2019	Q1	4,081,672	8.02%	327,350		0.34	4.22	–
2015	Q5	4,825,934	2.00%	96,519	26,582 (19.50%)	0.50	24.87	4.89
2019	Q5	5,150,654	2.39%	123,101		0.57	23.89	5.66

Column C: Population estimates obtained from the Australian Bureau of Statistics.

Column D: Percent prevalence of population with very-high psychological distress in each IRSD quintile for 2015 obtained from the 2014/15 Australian National Health Surveys; prevalences for 2019 obtained from the 2017/18 Australian National Health Survey.

Column E obtained by multiplying columns C and D.

Column F obtained by subtracting the 2019 data in columns D and E from the corresponding 2015 data, and then calculating the proportional percent increase.

Column G obtained by total number of services accessed by residents in the IRSD quintile areas in a year divided by the corresponding population number in column C.

Column H obtained by total number of services accessed by residents in the IRSD quintile areas in a year divided by the corresponding population number in column E.

Column I obtained for each year by the Equity-indicator-adjusted service rate (column H) in Q5 divided by that in Q1.

* Equity indicators close to 1 indicate relative equal distribution of needs-based services in the most and least disadvantaged quintiles. Whereas, equity indicators greater than 1 indicate more services accessed in the least disadvantaged areas despite having relative equal needs for services.

### Datasets and measures

The Medicare administrative dataset (Australian Institute of Health and Welfare, 2021b) contains all mental health-specific Medicare items claimed for services accessed in Australia between 2015 and 2019. The Australian Institute of Health and Welfare (AIHW) designated items as mental health-specific, categorising them into five provider groups: GPs, psychiatrists, clinical psychologists, other psychologists and accredited mental health social workers and occupational therapists (other allied health); AIHW documentation lists the item numbers (Australian Institute of Health and Welfare, 2023a). Medicare items were aggregated by Statistical Area 3 (SA3) geographic regions, a unit defined in the Australian Statistical Geography Standard (Australian Bureau of Statistics, 2021), a classification system dividing Australia into social-geographical areas (Australian Bureau of Statistics, 2021). SA3 is most often used for health data and regions typically contain 30,000–130,000 people.

The Australian National Health Surveys (NHS) are nationally representative, administered by trained interviewers, and conducted triennially by the Australian Bureau of Statistics (Australian Bureau of Statistics, 2023a). The Kessler Psychological Distress Scale 10 (K10) is collected in the NHS and is a measure for approximately capturing population mental health. Items relate to anxiety and affective disorder symptoms and have a good concordance with other disorders (Slade *et al.*, 2011). Unlike psychiatric diagnoses, the K10 is short, easily administered, and collected in sequential cross-sectional NHSs globally (Kessler *et al.*, 2009), enabling assessment of population trends in mental health (Enticott *et al.*, 2022). The K10 is also indicative of untreated or unresolved mental disorders, for example in those not accessing services, as it captures symptoms from the past 30 days (Enticott *et al.*, 2016).

Based on the Australian Bureau of Statistics scoring method, a threshold of ≥30 on the K10 (very-high levels of psychological distress) was used to estimate the prevalence of those with a mental health service need in the 2014/15 and 2017/18 NHSs. In previous Australian general population research, this threshold has expected high specificity (0.99) for currently active anxiety and affective disorders and high positive predictive value (PPV 89%) for 12-month mental disorders (Andrews and Slade, 2001). The Australian Bureau of Statistics high level distress categorisation of 22–29 was not included because the objective of universal healthcare is to target care to those who need it most. In the most recent 2022 national survey in Australia, ∼20% of the working-age population had high level and above distress, whilst 6% had very-high level distress. It is not feasible nor desirable to provide mental healthcare to 20% (1-in-5 people) of the population: it is expensive, likely to include many with subthreshold conditions, and could disenfranchise people from using other mechanisms such as self-help or online tools, which are recommended in guidelines for lower-level conditions. The commonly-used threshold of very-high distress was therefore expected to be the most valid proxy for relative levels of more severe mental health problems, representing those with the greatest need for care. According to the most recent evaluation of Better Access (Pirkis *et al.*, 2022), people with very high psychological distress form the largest category of Better Access service users (50%): they were also the group most likely to significantly improve from treatment and the least likely to show significant deterioration.

The Index of Relative Socioeconomic Deprivation (IRSD) is an area-based measure of socioeconomic disadvantage, represented as a numeric score, and calculated by the Australian Bureau of Statistics using several variables from the 2016 Census, including weekly household income, the highest level of education achieved, amongst others (Australian Bureau of Statistics, 2023b). We calculated a fractional-rank IRSD score (weighted by SA3 population) for each SA3, and used this to classify SA3s into quintiles by disadvantage, with IRSD Quintile 1 (Q1) representing the most disadvantaged areas and IRSD Quintile 5 (Q5) the least.

### Equity indicator calculation

We estimated the number with very-high psychological distress in each SA3 based on nationally estimated prevalences within IRSD quintiles (Enticott *et al.*, 2022). Per SA3, we divided the number of services claimed by the estimated number with mental healthcare need, producing equity-adjusted service use-rates. We averaged these rates across each IRSD quintile, producing a mean rate per quintile. The ratio of service use-rates between IRSD Q5/Q1 is the equity indicator (Table 2). An equity indicator of 1 represents the equal distribution of services amongst those with the greatest needs across IRSD Q1 and Q5. Larger values indicate that more services were accessed in Q5 than Q1, despite having relatively equal service needs.

*T*-tests compared the equity-adjusted service use-rates between different years, IRSD quintiles and providers. The significance level was set to *p* ≤0.001 to account for multiple comparisons (Lee and Lee, 2018). We calculated 95% confidence intervals for the standard normal distribution. To assess the distribution of mental health services across IRSD quintiles (Meadows *et al.*, 2015; Wagstaff and Neelsen, 2020), we also calculated a standard (Clarke’s) concentration index (Clarke *et al.*, 2002) using clustered standard errors at the SA3 level. The concentration index is commonly used to assess socioeconomic inequality in healthcare (Wagstaff and Neelsen, 2020). Values range from −1 to +1; greater positive values indicate that services are concentrated in the least disadvantaged areas. Greater negative values indicate that services are concentrated in the most disadvantaged areas.

## Results

### Subpopulation with greatest need for mental healthcare

The overall percentage of Australians with the greatest estimated mental healthcare need was 4.44% in 2014/15 and 5.05% in 2017/18. This estimate was 2.4% in residents of the least disadvantaged area quintile (Q5) and 8.0% in the most disadvantage area quintile (Q1) in 2017/18 (Table 1).

Table 2 presents the estimated numbers of those with the greatest mental healthcare need in Q1 and Q5. Between 2015 and 2019 the proportional population increase in those with an estimated mental healthcare need was 27.7% (Q1) and 19.5% (Q5).

### Mental health services utilisation per capita

**Figure 1.**
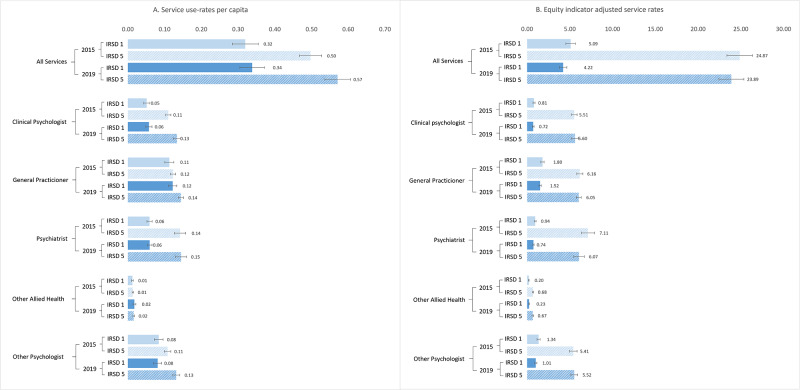
Comparison of 2015 and 2019 service rates by Index of Relative Socio-economic Disadvantage (IRSD) area quintiles 1 and 5. (a) Means are calculated by the number of services divided by the total population in the area. (b) Equity indicator adjusted service rates, indicating the rate of services usage in the subpopulation with greatest need for mental healthcare. All confidence intervals are standard normal 95% confidence intervals.

In total, 57,940,296 mental health specific service items were claimed under Medicare between 2015 and 2019. Table 3a and Fig. 1a show the mean service-use rates per capita.

**Table 3.** Equity analyses showing the IRSD 5:1 ratio (least disadvantaged Q5 service rates divided by most disadvantaged Q1 service rates), and concentration indexes. In 2019, the equity indicator adjusted IRSD 5:1 ratio demonstrate that Q1 areas received almost 6 times fewer services, and Q1 individuals with a need for care received 566% fewer services compared to their Q1 counterparts

			A. Australian services per capita				B. Equity indicator adjusted (services usage in the subpopulation with greatest need for mental healthcare)			
Service provider	Year	IRSD	Mean service use-rate per capita (95% CI)	SD	Ratio IRSD 5:1	Concentration index (SE)^n^	Mean service use-rate in subpopulation with need for care (95% CI)	SD	Equity indicator*	Concentration index (SE)
**All Services**	2015	IRSD 1	0.32 (0.28–0.36)***	0.15	1.56	0.1097 (0.0201)	5.09 (4.53–5.65)***	2.32	4.88	0.0942 (0.0235)
	2015	IRSD 5	0.5 (0.47–0.53)	0.12			24.87 (23.37–26.36)	6.13		
	2019	IRSD 1	0.34 (0.3–0.37)***	0.14	1.69	0.1309 (0.0197)	4.22 (3.8–4.64)***	1.73	5.66	0.1163 (0.0249)
	2019	IRSD 5	0.57 (0.54–0.61)	0.14			23.89 (22.44–25.35)	5.98		
**Clinical Psychologist**	2015	IRSD 1	0.05 (0.04–0.06)***	0.03	2.16	0.17 (0.0213)	0.81 (0.69–0.93)***	0.50	6.77	0.1581 (0.0263)
	2015	IRSD 5	0.11 (0.1–0.12)	0.03			5.51 (5.17–5.86)	1.42		
	2019	IRSD 1	0.06 (0.05–0.07)***	0.03	2.32	0.1889 (0.0213)	0.72 (0.61–0.82)***	0.43	7.80	0.1918 (0.0278)
	2019	IRSD 5	0.13 (0.12–0.14)	0.04			5.6 (5.18–6.01)	1.70		
**General Practitioner**	2015	IRSD 1	0.11 (0.1–0.13)	0.05	1.09	0.0446 (0.0194)	1.8 (1.6–2)***	0.82	3.42	0.0449 (0.0225)
	2015	IRSD 5	0.12 (0.12–0.13)	0.03			6.16 (5.81–6.52)	1.45		
	2019	IRSD 1	0.12 (0.11–0.13)***	0.05	1.18	0.0695 (0.019)	1.52 (1.38–1.66)***	0.58	3.97	0.0626 (0.0249)
	2019	IRSD 5	0.14 (0.14–0.15)	0.03			6.05 (5.75–6.35)	1.22		
**Psychiatrist**	2015	IRSD 1	0.06 (0.05–0.07)***	0.03	2.40	0.2023 (0.0253)	0.94 (0.83–1.06)***	0.47	7.53	0.1722 (0.0295)
	2015	IRSD 5	0.14 (0.13–0.16)	0.06			7.11 (6.36–7.87)	3.10		
	2019	IRSD 1	0.06 (0.05–0.07)***	0.03	2.44	0.2107 (0.0244)	0.74 (0.66–0.82)***	0.34	8.18	0.1879 (0.029)
	2019	IRSD 5	0.15 (0.13–0.16)	0.06			6.07 (5.45–6.7)	2.55		
**Other Allied Health**	2015	IRSD 1	0.01 (0.01–0.02)	0.01	1.08	0.0265 (0.0232)	0.2 (0.16–0.24)***	0.15	3.38	0.0136 (0.0262)
	2015	IRSD 5	0.01 (0.01–0.02)	0.01			0.68 (0.6–0.77)	0.34		
	2019	IRSD 1	0.02 (0.01–0.02)	0.02	0.88	0.00003 (0.0236)	0.23 (0.18–0.28)***	0.21	2.97	−0.0118 (0.03)
	2019	IRSD 5	0.02 (0.01–0.02)	0.01			0.67 (0.58–0.76)	0.38		
**Other Psychologist**	2015	IRSD 1	0.08 (0.07–0.1)***	0.05	1.28	0.0703 (0.0208)	1.34 (1.16–1.53)***	0.77	4.03	0.0562 (0.025)
	2015	IRSD 5	0.11 (0.1–0.12)	0.04			5.41 (4.97–5.85)	1.81		
	2019	IRSD 1	0.08 (0.07–0.09)***	0.05	1.62	0.1117 (0.0207)	1.01 (0.88–1.15)***	0.56	5.45	0.0919 (0.028)
	2019	IRSD 5	0.13 (0.12–0.14)	0.04			5.52 (5.12–5.92)	1.65		

*Notes*: SD = standard deviation; SE = standard error (robust); 95% CI = 95% confidence intervals.

*** indicates that *p* ≤ 0.001 in a *t*-test comparing the mean rate of service use in IRSD 1 vs IRSD 5 within the same year.

Concentration Indexes were calculated for 2015 and 2019 and assessed the distribution of services across the IRSD quintiles. *F*-tests compared the concentration indexes between 2015 and 2019, however no significant differences were found.

n Raw concentration index data is republished from a Research Letter (currently under review at The Medical Journal of Australia: Meadows et al.)

The mean total service-use rates increased from 2015 to 2019 (Table 3). This was non-significant for Q1 and significant for Q5 (service use-rates of 0.497 to 0.571, *p* = 0.0009). Mean rates increased across all providers from 2015 to 2019 in Q5 and Q1 but were only significant in Q5 for clinical psychologists (0.110 to 0.134, *p* = 0.0001), GPs (0.123 to 0.145, *p* ≤ 0.0001) and other psychologists (0.108 to 0.132, *p* = 0.0002).

See supplemental files for *T* and *F*-test result tables ranked by IRSD across all years.

### Equity-indicator-adjusted service rates

The equity-indicator-adjusted service rates, indicating the rate of services use in the sub-population with greatest need for mental healthcare, are shown in Table 3b and Fig. 1b.

Different trends emerged when examining changes in the mean service-use rates in the equity-indicator-adjusted service rates, as compared to services per capita. For example, there was a non-significant decrease for Q1 (service-use rates of 5.09 to 4.22) and Q5 (24.87 to 23.89). Time trends also varied by service provider. Non-significant decreased mean rates were apparent in Q1 for all providers excepting other allied health. Q5 saw non-significant increases (clinical psychologists, other psychologists) and decreases (GP, psychiatrist, other allied health) in mean rates over time (Table 3).

The findings for the 2, 3 and 4^th^ area disadvantage quintiles lie between those provided above, see Supplementary files.

### Equity indicators and IRSD 5:1 service ratios

Table 3 presents the Equity Indicators and the per capita IRSD 5:1 ratio of the mean service use-rates. Nearly all ratios were greater than 1, indicating greater mean service use-rates in Q5 than Q1. In 2019, the IRSD 5:1 ratio for the entire population was 1.69; those in Q5 received about 70% more mental health services in total as those in Q1.

The Equity Indicators represent the IRSD 5:1 for the sub-population with an estimated mental health service need. In 2019 it was 5.66 for all services, indicating that those in Q5 with a mental health need received nearly six times the services than their Q1 counterparts.

Overall Fig. 1 shows that between 2015 and 2019, the equity indicators were largest for psychiatrists, then clinical psychologists, other psychologists, GPs and other allied health professionals, in descending order.

### Equity analysis: concentration indices

Table 3 present concentration indexes assessing the distribution of mental health services across the IRSD quintiles in 2015 and 2019 (and the supplementally file has all years). The concentration index values significantly increased over time (indicating increased inequity) when examining the data from all years between 2015 and 2019 (significant *p* for trend). This increase was evident for services in totality, and for GPs, clinical psychologists, other psychologists and psychiatrists.

## Discussion

We developed a national mental health services equity indicator using robust national health data encompassing healthcare need, service utilization and socioeconomic status. This indicator allowed direct comparison between regions with varying healthcare needs within a country by representing service utilization rates, calculated for the subpopulation with the greatest need for care. Within the Australian health system context, from 2015 to 2019, the estimated proportion of those with the greatest mental healthcare need had increased for the most socioeconomically disadvantaged (6.3% to 8.0%) and the least disadvantaged (2.0% to 2.4%) quintiles. The socioeconomic disparity persisted, with the most disadvantaged having significantly lower service utilization (422 services per 100 people) compared to the least disadvantaged (2,389 services per 100 people), corresponding to an equity indicator of 5.7 (approximately a 6-fold difference). In other words, the equity indicator describes that for those with the greatest need for care, we have six times more services accessed by people who reside in the least compared to the most disadvantaged areas in Australia. Services with higher out-of-pocket costs, including psychiatrists and clinical psychologists, showed pronounced disparities with equity indicators of 8.2 and 7.8, respectively.

Equity indicators calculated for other countries using this method would produce different results across settings and regions globally. In Australia, we showed stark inequity has occurred within a publicly funded system aiming for universal healthcare, highlighting the need for considerable reform. The Australian federal government regularly releases accurate mental health service usage data, which have shown consistent increases. Medicare-subsidised mental health services grew from 6% of Australians in 2009/10 to 15% in 2020/21. However, these use-rates do not assess whether services have reached those in greatest need. Socioeconomic disparities in Australia parallel those in other nations, with a higher prevalence of mental health issues in deprived areas compared to affluent ones. In 2019, highly elevated psychological distress, indicative of severe mental illness, was at 8% in the most disadvantaged area quintile and 2% in the least disadvantaged quintile. Past Australian evaluations of Medicare mental health services were infrequent and labour-intensive, often involving data linkage and new samples. Initial evaluations from 2007/08 to 2009/10 reported small socioeconomic differences in service use per-person (Pirkis *et al.*, 2011) However, later analyses for the same period revealed larger socioeconomic disparities in the total number of services provided per area population, particularly for psychiatrist-provided services (Meadows *et al.*, 2015). A recent evaluation spanning 2018–2021, linking Medicare data with other sources, confirmed service inequities, particularly affecting individuals with low income despite their higher levels of need (Pirkis *et al.*, 2022). All previous evaluations reported service usage rates by area, unadjusted by area need. Instead, utilizing the equity indicator presented in this paper would have provided a straightforward method for evaluating equitable service usage across Australia, and provide benchmarking across areas on an annual basis.

Effective policy should ensure that evidence-based high-quality mental healthcare is provided based on need, particularly in socioeconomically disadvantaged areas (Patel *et al.*, 2018). For example, Australian policy makers have shown receptivity to national equity analyses, as evidenced by the use of concentration indexes to quantify socioeconomic inequity in new Medicare psychiatry services (Yeatman *et al.*, 2023). During the COVID-19 pandemic, the normalization of video-linked telehealth mental health services was expected to improve socioeconomic equity, but instead led to increased inequality, highlighting an unintended consequence (Yeatman *et al.*, 2023). This national equity analysis informed policy decisions, resulting in the maintenance of telephone-linked psychiatry services due to their lesser inequity (Yeatman *et al.*, 2023). Other mental health service planning approaches that consider socioeconomic disadvantage have historically been successful at the state-level in Australia (Kirigia, 2009; Meadows and Singh, 2003) and in England (Barr *et al.*, 2014). However, challenges remain in implementing health reforms following indicator surveillance (Johnston *et al.*, 2021).

Globally, equity-adjusted indicators for other areas of healthcare are established and considered crucial for improving outcomes (Wagstaff and Neelsen, 2020). These indicators incorporate characteristics such as the presence of a specific medical condition, age, sex, socioeconomic status, disability and population size to assess health service need (Radinmanesh *et al.*, 2021). The impact of these indicators on mental healthcare is less explored due to limited publications (Johnston *et al.*, 2021). Existing literature suggests that needs-based indicators can have mixed impacts on reducing inequity in health outcomes and access (Barr *et al.*, 2014; Johnston *et al.*, 2021). Challenges include ensuring resources are distributed based on need and not just service type or population size, and that measures to improve access benefit those with greater health needs (Johnston *et al.*, 2021; Kirigia, 2009; Yeatman *et al.*, 2023).

### Future work

Why does socioeconomic inequity in mental health services persist? Studies from high-income countries with universal healthcare (including Australia) suggest that those in socioeconomically disadvantaged areas may be less likely to access or receive evidence-based mental healthcare (Allison *et al.*, 2023; Giebel *et al.*, 2020). Mental health services are largely located in more affluent urban areas in Australia (Enticott *et al.*, 2016), and Medicare’s subsidy freeze since 2014 has led to increased out-of-pocket costs, potentially hindering access, particularly for those in disadvantaged areas (Rosenberg *et al.*, 2022). Implementing a national equity indicator for benchmarking service provision is crucial to guide interventions for those with the greatest need. Importantly, the equity indicator could be included within a suite of public health policy measures monitored by governments and population stakeholders. Since social and economic factors (e.g. poverty, housing insecurity and domestic violence) contribute to mental health disparities, improving access to mental healthcare is one part of a broader public policy approach (World Health Organization, 2014).

Implementing a national equity indicator is essential to differentiate where healthcare resources should be scaled based on the greatest need within areas. The equity indicator presented here could be vital to measure and report annually, aligning with the aim of improving population mental health in Australian mental health policy (Carbone, 2024; Commonwealth of Australia, 2021) and global initiatives (Patel *et al.*, 2018). It can seamlessly integrate into existing health monitoring systems, utilizing established national data sources. In cases where reliable national data is unavailable, indicators can be estimated from other survey data, as seen in comparative epidemiological work (Wagstaff and Neelsen, 2020). Tracking socioeconomic disparities in mental health service use can identify where the key principle of universal healthcare – equitable access for improving whole-of-population health – is not fully realised, enabling interventions to address this gap. In our Australian case study, the prevalence of mental disorders occurring in past year is now approximately one-in-four people in Australia. Providing mental healthcare to 20 or 25% of the Australian population each year is not feasible or desirable. Instead, services should be targeted to those with the greatest need, and other evidence-based effective approaches (such as self-help strategies or digital interventions) targeted to those with mild to moderate conditions (Carbone, 2024). While this is the main feature of the stepped-care or staged care models, variations in resource levels across settings and locations may mean that the application of such models also present challenges to the equitable delivery of care (LaLonde *et al.*, 2022; Meadows and Shawyer, 2021; Sawrikar *et al.*, 2021).

Countries can implement equivalent indicators, or establish national minimum datasets to track mental health needs and service utilization based on socioeconomic disadvantage and other determinants (Ribeiro *et al.*, 2017; World Health Organization, 2014). This derived indicator can inform service planning based on population needs and risk-adjusted factors.

### Strengths and limitations

Estimated need in our study was taken from NHS estimates of those with very-high level psychological distress scores, affecting 3.8% and 5.1% of working age (18–64 years) Australians (Enticott *et al.*, 2022). This is lower than the 17% estimated population rate of any mental health service use (Australian Bureau of Statistics, 2023a). However, as a case study to demonstrate the utility of an indicator to identify inequitable service gaps, the premise was to identify those with greatest needs (reflecting pragmatic use of available data). This threshold is likely the most comparable with more severe and disabling mental health need (Enticott *et al.*, 2022).

In Australia, services delivered by hospital and community mental health centres are not captured in Medicare data. However, a sensitivity analysis was conducted by including the number of community-based mental health services in addition to Medicare services in the indicator; the disparity in service use between the most and least disadvantaged quintiles remained although was reduced. See supplementary file for the sensitivity analysis and Table S2. Community mental health services in Australia are distinct from Medicare-subsidised mental health services, having a high entry-threshold, typically treating a client-base with very serious mental illness at times of acute illness, and complex clients (Australian Institute of Health and Welfare, 2021a; Cook, 2019; Health Direct, 2022). With often high caseloads, support to this group may be limited by time with limited workforce, and resources to provide adequate support (Box 1). Community mental health services are therefore not a ‘replacement’ for Medicare-subsidised mental health in socioeconomically disadvantaged areas, as this creates a two-tiered healthcare system contrary to the principle of universality.

Another Australian study reported concentration indexes and compared service use by income (Bartram and Stewart, 2019) however it is difficult to compare this study to ours as they had estimated concentration indexes using regression methods that adjusted for socio-demographic variables (education, immigration status, non-urban residence). Our concentration indexes were calculated on the actual number of services within areas, and our findings are further supported by their similarity to those calculated previously using the same socioeconomic area categorisation (Meadows *et al.*, 2015). Our findings further converge with those from a recent representative household sample that adjusted for an individual’s need, reporting significantly greater utilisation of psychiatry care in the advantaged and most advantaged area quintiles (Hashmi *et al.*, 2023). Our study is more robust, as we analysed a national dataset (not a sample); in addition, they adjusted for the individual factors of age and sex, whilst we did not, in order to produce an indicator that can be applied by policy makers to real-world geographic areas.

## Conclusion

We have generated a mental health equity indicator based on healthcare need, service use and socioeconomic status, demonstrating feasibility and in a national case study in Australia. This indicator has identified major inequity in the current mental healthcare system, informing communities, healthcare providers and policymakers on the imperative for system reform to achieve universal equitable healthcare access. It also holds the potential to monitor progress on reforms and to benchmark within countries over time and across. For example, this Australian case study provide a baseline before the COVID-19 pandemic and enable us to examine effects from later policy changes enacted during the pandemic and afterwards. This indicator could facilitate global country comparisons, as well as within regions.

## Supporting information

Dawadi et al. supplementary material 1 Dawadi et al. supplementary material

Dawadi et al. supplementary material 2 Dawadi et al. supplementary material

## Data Availability

Anonymised, individual participant data for the estimated resident population and IRSD of each SA3 (https://www.abs.gov.au/AUSSTATS/abs@.nsf/DetailsPage/2033.0.55.0012016?OpenDocument), and K10 scores are available from the Australian Bureau of Statistics (https://www.abs.gov.au/statistics/microdata-tablebuilder/available-microdata-tablebuilder/national-health-survey#previous-releases). Mental health service use data, aggregated by SA3, is available from the AIHW data custodian (https://www.aihw.gov.au/mental-health/resources/archived-content?&page=2). All authors confirm that they had full access to all the data in the study.

## Supplementary material

The supplementary material for this article can be found at https://doi.org/10.1017/S2045796024000738.
