# Supplementary material for: An equity indicator for assessing mental healthcare access: a national population case study
Source: Epidemiol Psychiatr Sci. 2024 Nov 29;33:e70. doi: 10.1017/S2045796024000738 (PMC11669800; doi:10.1017/S2045796024000738)
Supplement: Dawadi et al. supplementary material 1 — Dawadi et al. supplementary material [file S2045796024000738sup001.docx]

Contents

[Supplementary – Detailed methods 1](#_Toc174717581)

[Measures and data 2](#_Toc174717582)

[Healthcare need 2](#_Toc174717583)

[Service utilization 3](#_Toc174717584)

[Data linkage and creating the indicator 3](#_Toc174717585)

[Other information 4](#_Toc174717586)

[Sensitivity analysis: Community mental health services 5](#_Toc174717587)

[Supplementary Table S1 7](#_Toc174717588)

[Supplementary Table S2 14](#_Toc174717589)

[References: 15](#_Toc174717590)

# Supplementary – Detailed methods

We devised an equity indicator to monitor trends in the proportion of inhabitants with a mental health service need, who are using mental health services (1). The equity indicator was generated from three high-quality, publicly-available national data sources with information on healthcare need, service use and area-based socioeconomic status (Table 1). The indicator enables direct comparison between areas with difference care need profiles.

In our case study of Australia: First, we estimated the percentage of those with a mental healthcare need in each quintile of socioeconomic disadvantage using the Australian National Health Surveys. These values informed the equity indicator. Second, we reported mental health service utilisation by examining administrative health data (Medicare) (Australian Institute of Health and Welfare 2022). Finally, we calculated equity indicators and also equity indicator adjusted service rates and concentration indexes, and examined the socioeconomic disparities within area based quintiles.

While adjusting for socioeconomic variables is a common practice in epidemiological research, we found no examples in peer-reviewed literature that employed the below approach to report service utilization rates adjusted for area-based need for services. Although, this concept has been incorporated into resource distribution and service planning formulas worldwide (2, 3), we identified no instances of its use in generating a comprehensive equity indicator for national mental health services rates.

## Measures and data

### Healthcare need

Measuring health care need to compare countries is estimated from national household surveys (4), and gaps in this data can be supplemented with other survey data and (where necessary) non-survey data from other sources as is done in the World Bank's Health Equity and Financial Protection Indicators database (4).

The Australian National Health Surveys (NHS) are nationally representative, administered by trained interviewers, and conducted every three years by the Australian Bureau of Statistics (ABS). The response rate was 82% (comprising 19,259 Australians) for the 2014/15 NHS, and 76% (21,315 Australians) for the 2017/18 NHS, (5) (6). Data for the area-based Index of Relative Socioeconomic Disadvantage and the Kessler-10, were derived from the adult (18 to 64 years) population in the each NHS. The sub-population with a mental health service need was estimated using the prevalence of those reporting very-high levels of psychological distress in the 2014/15 and 2017/18 NHSs. Other countries without this information could develop the indicator using other measures of mental health need

The Kessler-10 (K10) psychological distress scale is collected in national health surveys globally (7), and is a measure for approximately capturing population mental health. The K10 is short, easily administered, and included in the NHS so trends can be assessed by examining sequential cross-sectional surveys (8). It captures symptoms from the past 30 days and thus is, also indicative of untreated or unresolved mental disorders, such as in those who are undiagnosed because they have not accessed mental health services (9).

The K10 contains ten items on a Likert-type scale. Items relate to anxiety and affective disorder symptoms and have a good concordance with other disorders (10). Total scores are obtained by summing item responses, and cut-offs are psychometrically established. Scores ≥30 are considered to be “very-high” level, having an overall 80% validity with any CIDI-diagnosed mental disorder, (10) and high specificity (0.99) for currently active anxiety and affective disorders (11). Specifically, from data in Table 5 in that paper, in relation to ‘very-high’ scores we calculated a Positive Predictive Value for any 12-month ICD-10 disorder of 89%.

Our rationale for using the rate of very-high psychological distress as a proxy for the proportion of the population in need of care is well-justified. This approach aligns with the socioeconomic gradient seen in the distribution of mental disorders across different regions (9), and it is correlated with mental disorder rates (8, 10).

The population percentage of very-high K10 scores in working age (18-64 years) Australians ranged between 3.8% and 5.1% from 2001 to 2018 (Enticott et al 2022), lower than the estimated population rate of any mental health service use (17.1%,(12) so does not constitute an estimate of overall level of need. We propose though that this choice of rate, being the one with closest comparability with rates of more severe and disabling mental health care problems as otherwise measured (13), is the best of the available choices from the NHS for estimating relative levels of population need. The Medicare dataset was not disaggregated by age, so the prevalence of very-high psychological distress in working age Australians was assumed to capture the socioeconomic gradient in mental health need for the entire Australian population. The working-age population comprises 60% of the total Australian population (14), and socioeconomic gradients are intergenerationally propagated.

### Service utilization

Service utilisation for the entire country should come from reliable sources. In some countries the reported service administrative data is of low quality and integrity (4). In these cases, it has been recommended that service utilisation estimates come from other, more reliable sources such as national household surveys that also have socioeconomic data to allow for examination by socioeconomic status (4).

In our case study of Australia, the national health care service utilisation data is of high quality and accuracy. In Australia the publicly available administrative Medicare dataset (15) comprises all mental health-specific Medicare-items claimed for services accessed in Australia between 2015 and 2019. This includes data from all adults and children with 7.8% of services for those aged under 12 years (16). The Australian Institute of Health and Welfare designated items as mental health-specific, and further categorised them into five provider groups: GPs, psychiatrists, clinical psychologists, other psychologists, and accredited mental health social workers and occupational therapists (other allied health); specific item numbers are listed in Table 1 and the AIHW data source documentation (17).

Medicare item claims were aggregated by Statistical Area 3 (SA3) geographic regions in the Medicare dataset. SA3s are defined in the Australian Statistical Geography Standard (ASGS) (18), a 4-level hierarchically nested, classification system dividing Australia into social-geographical areas based regional identities of increasing size (Australian Bureau of Statistics 2021). SA3 is most often used for health data and typically divides regions into populations of 30,000 - 130,000 people. The data released does not contain information such as age or sex of consumers. More granulated data is not made publicly available, and the disaggregated data applied in the 2022 Better Access evaluation (19) is not made available to other researchers.

Socioeconomic status

National surveys or national Census are often good sources of socioeconomic data to allow for disaggregation by socioeconomic status within a country.

In our case study of Australia, the Index of Relative Socioeconomic Deprivation (IRSD) is an area-based measure of socioeconomic disadvantage calculated by the Australian Bureau of Statistics using several variables from the 2016 Census, including weekly household income, and the number of people with post-secondary qualifications, amongst others (20). We calculated a fractional-rank IRSD score (weighted by SA3 population) for each SA3 and used this to rank SA3 areas by disadvantage. In the current study IRSD Quintile 1 (Q1) represents the most disadvantaged areas and IRSD Quintile 5 (Q5) the least.

### Data linkage and creating the indicator

For our case study of Australia, Table 1 in the main manuscript shows the data sources linked for each year of service data. Since the Medicare dataset was aggregated at the SA3 level, we linked it to the population of each SA3 obtained from census data (21). Each SA3 was assigned to an IRSD quintile, using the SA3 population-weighted approach described above. Each SA3 was also assigned an estimated prevalence of people with very high K10, based on the nationally estimated rates in IRSD quintiles. For Medicare service data from 2015 and 2016, very high K10 rates were extracted from the 2014/15 NHS; for data from 2017, 2018, and 2019, very high K10 rates were extracted from the 2017/18 NHS.

2.2. Data analysis

For the case study of Australia, per capita service rates are calculated by: dividing the number of services by the population. Equity indicator adjusted service rates are calculated by: dividing the number of services by the subpopulation with need for mental healthcare. Socioeconomic disparities within areas are analysed using IRSD area-based quintiles. See Table 1 for data sources.

We calculated the Medicare mental health service-use rate per SA3 population, and averaged these rates across each IRSD quintile, producing a mean rate. The estimated mean service-use rate in the sub-population with a need for mental healthcare was also calculated for each IRSD quintile using a similar approach. All rates are expressed as a mean of SA3 service use-rates.

Equity analyses: IRSD 5:1 ratios were then calculated by dividing the mean service use-rate in IRSD quintile 5 (least disadvantaged) by the mean service use-rate in IRSD quintile 1 (most disadvantaged). The Equity Indictor calculation is shown in Table 2.

Additionally, we calculated a concentration index assessing the distribution of mental health services across the IRSD quintiles (4, 22). The concentration index is commonly used to assess socioeconomic inequality in health care (4). Values range from -1 to +1; greater positive values indicate that services are concentrated in the least disadvantaged areas. Greater negative values indicate that services are concentrated in the most disadvantaged areas.

T-tests compared the mean service use-rates between different years, IRSD quintiles, and providers. The significance level was set to p≤0.001 to account for multiple comparisons (23).

## Other information

The Medicare service data are not linked to mental health outcomes, nor disaggregated by age or sex, and the indicator uses an area-based (not-individual) measure of socioeconomic disadvantage. However, these are not limitations because the aim of the indicator was to provide practical information to policy-makers about area-based mental health service need and use; the focus of this case study is equity across groups.

Our rationale for using the rate of very-high psychological distress as a proxy for the proportion of the population in need of care is well-justified. This approach aligns with the socioeconomic gradient seen in the distribution of mental disorders across different regions (9), and it is correlated with mental disorder rates (8, 10).

# Sensitivity analysis: Community mental health services

Community mental health services are funded by the state governments, and are a distinct system from Medicare mental health services, which are federally funded. Community services comprise a service contact between a specialised community mental health care providers and a patient, and care is provided in a community or hospital-based outpatient care setting; the specific type of service and care team varies by state (24). Community mental health services have a high entry-threshold, typically treating a client-base with very serious mental illness at the times when illness suddenly worsens (24, 25). Caseloads include those experiencing acute episodes; complex clients; early psychosis; and clients with acquired brain injury (26). With often high caseloads, support provided to this group may be limited by time with limited workforce and resources.

Method for the sensitivity analysis

We obtained the number of community mental health services delivered per SA3 from the Australian Institute of Health and Welfare (25). We re-calculated the indicator using the number of Medicare and community mental health services to derive our service use rates (Table S2). The main analysis used the number of Medicare mental health services only.

Results from the sensitivity analysis

Table S2 shows the distribution of community mental health services is relatively equitable across the most and least disadvantaged IRSD quintiles, with equity indicator values of 1.31 in 2015 and 1.53 in 2019. However, the disparity between quintiles remained when both Medicare and community mental health services were considered together, with an equity indicator of 2.55 in 2015 and 3.02 in 2019.

Discussion and interpretation of the sensitivity analysis

When considering mental health care services for those that have the greatest need for care, it is important to consider the equity indicator when including both Community mental health services and Medicare-subsidised mental healthcare. The findings show that in 2019 the Australian equity indicator of 6 for Medicare-subsidised mental health care, reduces to 3 when additionally including Community mental health services. In other words, the equity indicator describes that for those with the greatest need for care, we have six times more Medicare-subsidised mental healthcare services accessed by people who reside in the least compared to the most disadvantaged areas in Australia. When including Community mental health services, the equity indicator describes three times more services accessed by people with the greatest need for care who reside in the least compared to the most disadvantaged areas in Australia.

Although it appears the Community mental health services are partially mitigating the socioeconomic disparity in Medicare-subsidised mental health services; the two service types cannot been viewed as equal. As outlined above, community mental health services have a high entry-threshold, typically treating a client-base with serious mental illness in times of acute illness and complex clients (25), and since staff in these services often have high caseloads, support to this group may be limited by time, workforce, and resources. Community mental health services are therefore not a “replacement” for Medicare-subsidised mental health care in socioeconomically disadvantaged areas, as the two services are substantially different, and this creates a two-tiered healthcare system contrary to the principle of universality.

## Supplementary Table S1

Table S1: The list of MBS mental health items included in the Medicare Dataset. Table reproduced from the Australian Institute of Health and Welfare (17)

| **Provider type** | **Item group** | **MBS Group** | **MBS item numbers** |
| --- | --- | --- | --- |
|  |  |  |  |
| Psychiatrists | Initial consultation new patient | Group A08 | 296, 297, 299, 134(a) |
|  |  |  |  |
|  |  | Group A40 (T) | 92437, 92466(a), 92477(a), 92506(a) |
|  |  |  |  |
|  | Patient attendances | Group A08 | 291, 293, 300, 302, 304, 306, 308, 310, 312, 314, 316, 318, 319, 320, 322, 324, 326, 328, 330, 332, 334, 336, 338, 136(a), 138(a), 140(a), 142(a), 144(a), 146(a), 148(a), 150(a), 152(a), 294(T), 288(T)(a), 353(T)(a), 355(T)(a), 356(T)(a), 357(T)(a), 358(T)(a), 359(T)(a), 361(T)(a), 364(a), 366(a), 367(a), 369(a), 370(a) |
|  |  |  |  |
|  |  | Group A40 (T) | 91827, 91828, 91829, 91830, 91831, 91837, 91838, 91839, 91840(a), 91841(a), 92435, 92436, 92461(a), 92462(a), 92463(a), 92464(a), 92465(a), 92475(a), 92476(a), 92501(a), 92502(a), 92503(a), 92504(a), 92505(a) |
|  |  |  |  |
|  | Group psychotherapy | Group A08 | 342, 344, 346, 154(a), 155(a), 156(a) |
|  |  |  |  |
|  |  | Group A40 (T) | 92455, 92456, 92457, 92495(a), 92496(a), 92497(a) |
|  |  |  |  |
|  | Interview with non‑patient | Group A08 | 348, 350, 352, 157(a), 158(a), 159(a) |
|  |  |  |  |
|  |  | Group A40 (T) | 92458, 92459, 92460, 92498(a), 92499(a), 92500(a) |
|  |  |  |  |
|  | Case conferencing | Group A15 | 855, 857, 858, 861, 864, 866 |
|  |  |  |  |
|  | Electroconvulsive therapy | Group T01 | 14224, 153(a), 340(a), 886(a) |
|  |  |  |  |
|  | Assessment and treatment of pervasive developmental disorder (PDD) | Group A08 | 289 |
|  |  |  |  |
|  |  | Group A40 (T) | 92434, 92474(a) |
|  |  |  |  |
|  | Repetitive Transcranial Magnetic Stimulation (rTMS) | Group T01 | 14216, 14217, 14219, 14220 |
|  |  |  |  |
|  | Eating Disorders Treatment Plan preparation and review | Group A36 | 90260, 90262(T)(a), 90266, 90268(T)(a) |
|  |  |  |  |
|  |  | Group A40 (T) | 92162, 92166(a), 92172, 92178(a) |
|  |  |  |  |
| General practitioners | Mental Health Treatment Plan preparation and review | Group A07 | 281, 282, 272, 276, 277 |
|  |  |  |  |
|  |  | Group A20 | 2715, 2717, 2710(a), 2700, 2701, 2702(a), 2712, 2719(a) |
|  |  |  |  |
|  |  | Group A40 (T) | 92116, 92117, 92122, 92123, 92128(a), 92129(a), 92134(a), 92135(a), 92112, 92113, 92118, 92119, 92124(a), 92125(a), 92130(a), 92131(a), 92114, 92126, 92120, 92132 |
|  |  |  |  |
|  |  | Group A42 | 93402(a), 93403(a), 93406(T)(a), 93407(T)(a), 93410(T)(a), 93411(T)(a), 93433(a), 93434(a), 93437(T)(a), 93438(T)(a), 93441(T)(a), 93442(T)(a), 93400(a), 93401(a), 93404(T)(a), 93405(T)(a), 93408(T)(a), 93409(T)(a), 93431(a), 93432(a), 93435(T)(a), 93436(T)(a), 93439(T)(a), 93440(T)(a), 93421(a), 93422(T)(a), 93423(T)(a), 93451(a), 93452(T)(a), 93453(T)(a) |
|  |  |  |  |
|  | Mental Health Treatment service | Group A07 | 279, 894(T)(a), 896(T)(a), 898(T)(a) |
|  |  |  |  |
|  |  | Group A20 | 2713 |
|  |  |  |  |
|  |  | Group A30 | 2121(a), 2150(a), 2196(a) |
|  |  |  |  |
|  |  | Group A40 (T) | 92115, 92127, 92121, 92133 |
|  |  |  |  |
|  | Eating Disorder Treatment Plan preparation, review and service | Group A36 | 90250, 90251, 90252, 90253, 90254, 90255, 90256, 90257, 90264, 90265, 90271, 90272, 90273, 90274, 90275, 90276, 90277, 90278, 90279(T)(a), 90280(T)(a), 90281(T)(a), 90282(T)(a) |
|  |  |  |  |
|  |  | Group A40 (T) | 92146, 92147, 92148, 92149, 92150, 92151, 92152, 92153, 92154(a), 92155(a), 92156(a), 92157(a), 92158(a), 92159(a), 92160(a), 92161(a), 92170, 92171, 92176, 92177, 92182, 92184, 92186, 92188, 92194, 92196, 92198, 92200 |
|  |  |  |  |
|  | Focused Psychological Strategies | Group A07 | 283, 285, 286, 287, 371(a), 372(a), 941(a), 942(a) |
|  |  |  |  |
|  |  | Group A20 | 2721, 2723, 2725, 2727, 2729(T)(a), 2731(T)(a), 2733(a), 2735(a) |
|  |  |  |  |
|  |  | Group A39 | 91283(a), 91285(a), 91286(a), 91287(a), 91371(T)(a), 91372(T)(a), 91721(a), 91723(a), 91725(a), 91727(a), 91729(T)(a), 91731(T)(a) |
|  |  |  |  |
|  |  | Group A40 (T) | 91818, 91819, 91820, 91821, 91842, 91843, 91844, 91845 |
|  |  |  |  |
|  |  | Group A41 | 93300(a), 93301(T)(a), 93302(T)(a), 93303(a), 93304(T)(a), 93305(T)(a), 93306(a), 93307(T)(a), 93308(T)(a), 93309(a), 93310(T)(a), 93311(T)(a), 93287(a), 93288(a), 93291(a), 93292(a) |
|  |  |  |  |
|  | Family Group Therapy | Group A06 | 170, 171, 172, 996(a), 997(a), 998(a) |
|  |  |  |  |
|  |  | Group A07 | 221, 222, 223 |
|  |  |  |  |
|  | Electroconvulsive therapy | Group T10 | 20104 |
|  |  |  |  |
|  | 3 Step Mental Health Process | Group A18 | 2574(a), 2575(a), 2577(a), 2578(a) |
|  |  |  |  |
|  |  | Group A19 | 2704(a), 2705(a), 2707(a), 2708(a) |
|  |  |  |  |
| Clinical psychologists | Psychological Therapy Services | Group M06 | 80000, 80001(T)(a), 80005, 80010, 80011(T)(a), 80015, 80020, 80021(T) |
|  |  |  |  |
|  |  | Group M17 | 91000(a), 91001(a), 91005(a), 91010(a), 91011(a), 91015(a) |
|  |  |  |  |
|  |  | Group M18 (T) | 91166, 91167, 91181, 91182 |
|  |  |  |  |
|  |  | Group M25 | 93312(a), 93313(a), 93330(a), 93331(T)(a), 93332(T)(a), 93333(a), 93334(T)(a), 93335(T)(a) |
|  |  |  |  |
|  |  | Group M27 | 93375(a), 93376(a) |
|  |  |  |  |
|  | Eating Disorder Psychological Treatment Service | Group M16 | 82352, 82353(T)(a), 82354, 82355, 82356(T)(a), 82357, 82358, 82359 |
|  |  |  |  |
|  |  | Group M18 (T) | 93076, 93079, 93110, 93113 |
|  |  |  |  |
| Psychologists including clinical psychologists | Enhanced Primary Care | Group M03 | 10968 |
|  | Focused Psychological Strategies | Group M07 | 80100, 80101(T)(a), 80105, 80110, 80111(T)(a), 80115, 80120, 80121(T) |
|  |  |  |  |
|  |  | Group M17 | 91100(a), 91101(T)(a), 91105(a), 91110(a), 91111(T)(a), 91115(a) |
|  |  |  |  |
|  |  | Group M18 (T) | 91169, 91170, 91183, 91184 |
|  |  |  |  |
|  |  | Group M26 | 93350(a), 93351(T)(a), 93352(T)(a), 93353(a), 93354(T)(a), 93355(T)(a), 93316(a), 93319(a) |
|  |  |  |  |
|  |  | Group M28 | 93381(a), 93382(a) |
|  |  |  |  |
|  | Psychology health service | Group M11 | 81355 |
|  |  |  |  |
|  |  | Group M29 | 93512(a), 93535(a) |
|  |  |  |  |
|  |  | Group M30 | 93557(a), 93590(a) |
|  |  |  |  |
|  | Assessment and treatment of PDD | Group M10 | 82000, 82015 |
|  |  |  |  |
|  |  | Group M18 (T) | 93032, 93035, 93040, 93043 |
|  |  |  |  |
|  | Eating Disorder Psychological Treatment Service | Group M16 | 82360, 82361(T)(a), 82362, 82363, 82364(T)(a), 82365, 82366, 82367(T) |
|  |  |  |  |
|  |  | Group M18 (T) | 93084, 93087, 93118, 93121 |
|  |  |  |  |
| Allied health providers | Enhanced Primary Care | Group M03 | 10956 |
|  |  |  |  |
|  | Focussed Psychological Strategies | Group M07 | 80125, 80126(T)(a), 80130, 80135, 80136(T)(a), 80140, 80145, 80146(T), 80150, 80151(T)(a), 80155, 80160, 80161(T)(a), 80165, 80170, 80171(T) |
|  |  |  |  |
|  |  | Group M17 | 91125(a), 91126(T)(a), 91130(a), 91135(a), 91136(T)(a), 91140(a), 91150(a), 91151(T)(a), 91155(a), 91160(a), 91161(T)(a), 91165(a) |
|  |  |  |  |
|  |  | Group M18 (T) | 91172, 91173, 91185, 91186, 91175, 91176, 91187, 91188 |
|  |  |  |  |
|  |  | Group M26 | 93356(a), 93357(T)(a), 93358(T)(a), 93359(a), 93360(T)(a), 93361(T)(a), 93362(a), 93363(T)(a), 93364(T)(a), 93365(a), 93366(T)(a), 93367(T)(a), 93326(a), 93327(a), 93322(a), 93323(a) |
|  |  |  |  |
|  |  | Group M28 | 93385(a), 93386(a), 93383(a), 93384(a) |
|  |  |  |  |
|  | Mental Health service | Group M11 | 81325 |
|  |  |  |  |
|  |  | Group M29 | 93506(a), 93529(a) |
|  |  |  |  |
|  |  | Group M30 | 93551(a), 93584(a) |
|  |  |  |  |
|  | Eating Disorder Treatment Service | Group M16 | 82350, 82351(T)(a), 82368, 82369(T)(a), 82370, 82371, 82372(T)(a), 82373, 82374, 82375(T), 82376, 82377(T)(a), 82378, 82379, 82380(T)(a), 82381, 82382, 82383(T) |
|  |  |  |  |
|  |  | Group M18 (T) | 93074, 93092, 93095, 93100, 93103, 93108, 93126, 93129, 93134, 93137 |
|  |  |  |  |
| Paediatrician | Eating Disorder Treatment Plan preparation and review | Group A36 | 90261, 90263(T)(a), 90267, 90269(T)(a) |
|  |  |  |  |
|  |  | Group A40 (T) | 92163, 92167(a), 92173, 92179(a) |
|  |  |  |  |
| a) Item discontinued  (T) Telehealth item  Provider type important notes:  General practitioner includes services provided by General practitioners and other medical practitioners, but excluding specialists or consultant physicians.  Clinical psychologist includes item numbers that can only be claimed by eligible Clinical psychologists.  Other psychologist includes item numbers that can be claimed by any eligible psychologist, clinical and other. The proportion of activity claimed against these items by Clinical psychologists has not been estimated in the presented data. That is, the services rendered by Clinical psychologists will be present in both the Clinical psychologist and Other psychologist categories. | | | |

## Supplementary Table S2

Table S2:Sensitivity analysis calculating equity-indicator-adjusted service use rates for community mental health services (CMH)

|  |  |  | |  |  | |  | **All service use-rate (per capita)** | | | **Equity indicator-adjusted service rate** | | |
| --- | --- | --- | --- | --- | --- | --- | --- | --- | --- | --- | --- | --- | --- |
| **Year** | **IRSD** | **C. MBS Services** | **D. CMH Services** | **E. All Services** | **F. Population** | **G. Percent with very-high psychological distress** | **H. Estimated number with greatest mental healthcare need** | **I. MBS service rate** | **J. CMH service rate** | **K. All services rate** | **L. MBS service rate** | **M. CMH service rate** | **N. All services rate** |
| 2015 | 1 | 1,382,222 | 2,197,982 | 3,580,204 | 3,904,729 | 6.28% | 245,217 | 0.3540 | 0.5629 | 0.9169 | 5.64 | 8.96 | 14.60 |
| 2015 | 5 | 2,451,573 | 1,137,397 | 3,588,970 | 4,825,934 | 2.00% | 96,519 | 0.5080 | 0.2357 | 0.7437 | 25.40 | 11.78 | 37.18 |
|  | |  |  |  |  |  | **Equity indicator (IRSD 5:1 Ratio)** | **1.44** | **0.42** | **0.81** | **4.51** | **1.31** | **2.55** |
|  |  |  |  |  |  |  |  |  |  |  |  |  |  |
| **Year** | **IRSD** | **MBS Services** | **CMH Services** | **All Services** | **Population** | **Percent with very-high psychological distress** | **Estimated number with greatest mental healthcare need** | **MBS service rate** | **CMH service rate** | **All services rate** | **MBS service rate** | **CMH service rate** | **All services rate** |
| 2019 | 1 | 1,500,752 | 2,330,501 | 3,831,253 | 4,081,672 | 8.02% | 327,350 | 0.3677 | 0.5710 | 0.9386 | 4.58 | 7.12 | 11.70 |
| 2019 | 5 | 3,013,508 | 1,336,612 | 4,350,120 | 5,150,654 | 2.39% | 123,101 | 0.5851 | 0.2595 | 0.8446 | 24.48 | 10.86 | 35.34 |
|  | |  |  |  |  |  | **Equity indicator (IRSD 5:1 Ratio)** | **1.59** | **0.45** | **0.90** | **5.34** | **1.53** | **3.02** |
| Notes:  MBS = Medicare Benefits Scheme (Medicare mental health services)  CMH = Community mental health service. Services comprise a service contact between a specialised community mental health care providers and a patient, and care is provided in a community setting. Service providers are government-funded. These data encompass all of Australia, and are obtained from the National Community Mental Healthcare Database (https://meteor.aihw.gov.au/content/699975). | | | | | | | | | | | | | |
| The all service-use rates in columns I, J, and K were calculated by dividing the services (columns C, D, and E, respectively) by the population (column F). Likewise, the equity-indicator-adjusted rates in columns L, M, and N were calculated by dividing the services (columns C, D, and E, respectively) by the estimated number with the greatest mental healthcare need (column H).  The MBS rates presented in the sensitivity analysis are slightly different from those presented in the main manuscript because SA3-Level data were not available for Community Mental Health services. For easier comparison in this sensitivity analysis, both MBS and CMH service-use-rates have been calculated by simple division. | | | | | | | | | | | | | |

# References:

1. Chandra PS, Chand P. Towards a new era for mental health. The Lancet. 2018;392(10157):1495-7.

2. Radinmanesh M, Ebadifard Azar F, Aghaei Hashjin A, Najafi B, Majdzadeh R. A review of appropriate indicators for need-based financial resource allocation in health systems. BMC Health Serv Res. 2021;21(1):674.

3. Rush B, Tremblay J, Babor TF. Needs-Based Planning for Substance Use Treatment Systems: The New Generation of Principles, Methods, and Models. Journal of Studies on Alcohol and Drugs, Supplement. 2019(s18):5-8.

4. Wagstaff A, Neelsen S. A comprehensive assessment of universal health coverage in 111 countries: a retrospective observational study. Lancet Glob Health. 2020;8(1):e39-e49.

5. Australian Bureau of Statistics. National Health Survey: First Results, 2014-15 In: Statistics ABo, editor. 2015.

6. Australian Bureau of Statistics. National Health Survey: First results. In: Statistics ABo, editor. 2018.

7. Kessler RC, Aguilar-Gaxiola S, Alonso J, Chatterji S, Lee S, Üstün TB. The WHO world mental health (WMH) surveys. Die Psychiatrie. 2009;6(01):5-9.

8. Enticott J, Dawadi S, Shawyer F, Inder B, Fossey E, Teede H, et al. Mental Health in Australia: Psychological Distress Reported in Six Consecutive Cross-Sectional National Surveys From 2001 to 2018. Frontiers in psychiatry. 2022;13.

9. Enticott JC, Meadows GN, Shawyer F, Inder B, Patten S. Mental disorders and distress: associations with demographics, remoteness and socioeconomic deprivation of area of residence across Australia. Australian & New Zealand Journal of Psychiatry. 2016;50(12):1169-79.

10. Slade T, Grove R, Burgess P. Kessler psychological distress scale: normative data from the 2007 Australian National Survey of Mental Health and Wellbeing. Australian & New Zealand Journal of Psychiatry. 2011;45(4):308-16.

11. Andrews G, Slade T. Interpreting scores on the Kessler Psychological Distress Scale (K10). Aust N Z J Public Health. 2001;25(6):494-7.

12. ABS. National Study of Mental Health and Wellbeing2023 [cited 2023. Available from: <https://www.abs.gov.au/statistics/health/mental-health/national-study-mental-health-and-wellbeing/2020-2022#cite-window1>.

13. Enticott J, Dawadi S, Shawyer F, Inder B, Fossey E, Teede H, et al. Mental Health in Australia: Psychological Distress Reported in Six Consecutive Cross-Sectional National Surveys From 2001 to 2018. Frontiers in Psychiatry. 2022;13.

14. Australian Bureau of Statistics. Twenty years of population change. Canberra; 2020.

15. Data Tables: Medicare-subsidised mental health-specific services 2019-20 [Internet]. <https://www.aihw.gov.au/mental-health/resources/archived-content?&page=2>. 2021 [cited 2021]. Available from: <https://www.aihw.gov.au/mental-health/resources/archived-content?&page=2>.

16. Australian Institute of Health and Welfare. Medicare-subsidised mental health-specific services 2023. Available from: <https://www.aihw.gov.au/mental-health/topic-areas/medicare-subsidised-services#Mental-health-specific-services>.

17. Medicare-subsidised mental health-specific services - data surce [Internet]. 2023 [cited 2022]. Available from: <https://www.aihw.gov.au/mental-health/topic-areas/medicare-subsidised-services#Data-source>.

18. Australian Bureau of Statistics. Australian Statistical Geography Standard (ASGS) Edition 3. In: Statistics ABo, editor. 2021.

19. Jane Pirkis, Dianne Currier, Meredith Harris, Mihalopoul C. Evaluation of the Better Access initiative – final report. University of Melbourne. 2022.

20. Australian Bureau of Statistics. IRSD 2018. 2018.

21. Australian Bureau of Statistics. Regional Population - Data Downloads. In: Statistics TABo, editor. 2022.

22. Meadows GN, Enticott JC, Inder B, Russell GM, Gurr R. Better access to mental health care and the failure of the Medicare principle of universality. Medical Journal of Australia. 2015;202(4):190-4.

23. Lee S, Lee DK. What is the proper way to apply the multiple comparison test? Korean journal of anesthesiology. 2018;71(5):353-60.

24. Cook L. Mental Health in Australia: a Quick Guide2019. Available from: <https://www.aph.gov.au/About_Parliament/Parliamentary_Departments/Parliamentary_Library/pubs/rp/rp1819/Quick_Guides/MentalHealth>.

25. Australian Institute of Health and Welfare. Community mental health care NMDS 2019–20: METEOR - metadata online registry; 2021 [Available from: <https://meteor.aihw.gov.au/content/699975>.

26. Direct H. Australian mental health services 2022 [Available from: <https://www.healthdirect.gov.au/australian-mental-health-services>.
